# Transition Metal‐Promoted V_2_CO_2_ (MXenes): A New and Highly Active Catalyst for Hydrogen Evolution Reaction

**DOI:** 10.1002/advs.201600180

**Published:** 2016-06-28

**Authors:** Chongyi Ling, Li Shi, Yixin Ouyang, Qian Chen, Jinlan Wang

**Affiliations:** ^1^ Department of PhysicsSoutheast UniversityNanjing211189China; ^2^ Synergetic Innovation Center for Quantum Effects and Applications (SICQEA)Hunan Normal UniversityChangsha410081China

**Keywords:** first principle, hydrogen evolution reaction, MXenes, vanadium carbides

## Abstract

Developing alternatives to precious Pt for hydrogen production from water splitting is central to the area of renewable energy. This work predicts extremely high catalytic activity of transition metal (Fe, Co, and Ni) promoted two‐dimensional MXenes, fully oxidized vanadium carbides (V_2_CO_2_), for hydrogen evolution reaction (HER). The first‐principle calculations show that the introduction of transition metal can greatly weaken the strong binding between hydrogen and oxygen and engineer the hydrogen adsorption free energy to the optimal value ≈0 eV by choosing the suitable type and coverage of the promoters as well as the active sites. Strain engineering on the performance of transition metal promoted V_2_CO_2_ further reveals that the excellent HER activities can maintain well while those poor ones can be modulated to be highly active. This study provides new possibilities for cost‐effective alternatives to Pt in HER and for the application of 2D MXenes.

## 1 Introduction

Hydrogen has been considered to be one of the most important candidates for the energy source of the next generation,^1^, ^2^ owing to the high energy density and environmentally friendly combustion product (H_2_O). Hydrogen evolution from electrocatalytic water splitting is one of the most efficient ways, where an ideal catalyst would be the key factor to the production of hydrogen. Precious metal platinum (Pt) is the most popular electrocatalyst for hydrogen evolution reaction (HER).^3^ However, the high cost and the insufficiency of Pt greatly hamper their practical utilization. To assure a sustainable hydrogen generation, tremendous efforts have been made to develop the earth abundant and cost‐effective alternatives to Pt in the past few decades, including non‐precious metal alloys, metal chalcogenides, metal carbides, metal nitrides, metal phosphides, and so on.^3^, ^4^, ^5^, ^6^, ^7^, ^8^ Among these alternatives, 2D layered materials (such as MoS_2_) have gained broad interest recently because of their extremely large surface areas, low cost, and excellent catalytic activity.^9^, ^10^, ^11^

Very recently, a new class of 2D layered materials with a general formula of M*_n_*_+1_X*_n_* labeled MXenes has been reported.^12^, ^13^, ^14^, ^15^, ^16^ Shortly after the synthesis of MXenes,^12^ their possible application as anode materials for Li ion batteries (LIBs) has been explored greatly and these MXenes exhibit good capability for high charge–discharge rates.^14^, ^17^, ^18^, ^19^ Besides LIBs, other applications, such as non‐Li‐ion batteries,^20^, ^21^ hydrogen storage,^22^, ^23^ supercapacitor,^24^ and thermoelectric materials,^25^ have also been investigated. MXenes were predicted to be potential heterogeneous catalysts owing to their high surface area and excellent thermostability as well.^26^ Recalling the fact that the release of H_2_ can always be observed during the synthesis process of MXenes,^12^ it is very possible that MXenes may be good catalyst for HER, which is never reported yet.

In this work, we select vanadium carbides with fully oxygen terminated surface (V_2_CO_2_) as the representative to investigate the HER performance by employing first‐principles calculations. Our results show that the strong hydrogen adsorption prohibits the pure V_2_CO_2_ as a potential HER catalyst. However, by introducing suitable transition metal atoms onto the surface, the H adsorption free energy can be tuned to be zero, comparable or even better than that of Pt (111) surface. The promotional effect is ascribed to the charge transfer between promoter atoms and surface O atoms. Moreover, the promotional HER performance shows good stability and can be further improved via external strain engineering.

## 2 Results and Discussion

Due to the high surface activity, all the MXenes produced to date are terminated by functional groups, such as OH, O, and F.^26^ The terminated groups (T) in MXenes have two energetically favorable orientations, resulting in three distinct structures:^25^ (i) the T groups are positioned above the top of X atoms, formed three T—M bonds with neighboring M atoms on both surfaces; (ii) the T groups are located above the hollow sites of M_3_X_3_ on both sides; (iii) T groups are above the top of X atoms on one surface and above the hollow sites of M_3_X_3_ on the other surface. Different MXenes and T groups have different ground state structures.^25^, ^26^ We considered all the possible absorption sites and found that the O atom favorably absorbs above the hollow site of C_3_V_3_. The stability of partial and fully oxidized V_2_C was also evaluated by computing the formation energies and the fully oxidized V_2_C is always thermodynamically most favorable when the chemical potential exceeds −7 eV, which corresponds to the ultralow oxygen partial pressure (see Figure S2a,b in the Supporting Information for more details). The stability of fully oxidized vanadium carbides is further evaluated by ab initio molecular dynamics simulation. As shown in Figure S2c (Supporting Information), the structure remains well even at a temperature of 1000 K, indicative of the high thermodynamic stability of V_2_CO_2_. Therefore, fully oxidized vanadium carbides (V_2_CO_2_) is selected as the study prototype in this work.

We first study the HER catalytic activity of pure V_2_CO_2_ by computing the reaction free energy of hydrogen adsorption at different hydrogen coverage (12.5%, 16.7%, and 25% monolayer (ML) H coverage on one surface), where the H atom prefers to adsorb on the top site of surface O atoms. The calculated Δ*G*_H_ is −0.45, −0.42, and −0.37 eV for 12.5%, 16.7%, and 25% ML H coverage (see Figure S1, Supporting Information), respectively, indicative of the strong interaction between H and surface O. Thus, the pure V_2_CO_2_ is not a good catalyst for HER activity.

**Figure 1:**
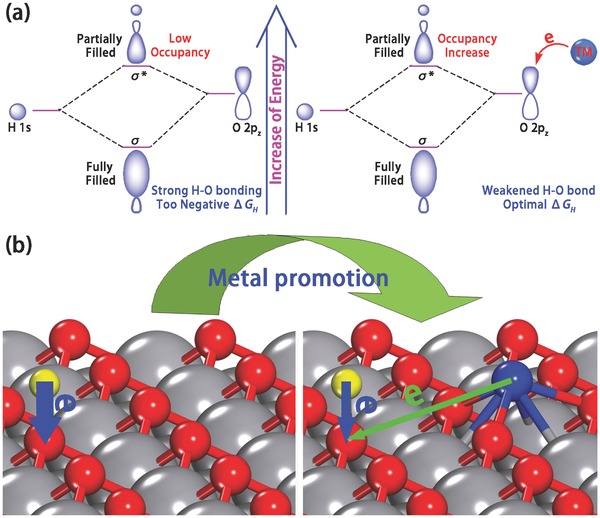
The scheme of modulating the HER performance of V_2_CO_2_ by introducing transition metal onto the surface. a) The combination of H 1s orbital and O 2p_z_ orbital forms a fully filled bonding orbital and a partially filled anti‐bonding orbital, in which the occupancy of anti‐bonding orbital will determine the strength of H—O bond. b) Charge transfer from H to O will occur when H adsorbs on O; by introducing a TM atom onto the surface, O will gain extra electrons from TM, leading to less charge transfer from H to O and a higher occupancy of anti‐bonding orbital when forming the H—O bond.

Since the high H binding strength causes the poor performance of V_2_CO_2_ in HER, it is natural to seek the way to weaken the interaction between H and O to improve the HER activity. In fact, when H adsorbs on surface O atom, the combination of H 1s orbital and O 2p_z_ orbital will form a fully filled bonding orbital (*σ*) and a partially filled anti‐bonding orbital (σ*). According to molecular orbital theory, the H bonding strength is determined by the occupancy of the partially filled anti‐bonding orbital, that is, the higher σ* occupancy, the weaker binding strength. So if we can introduce an electron donor (such as metal atoms) onto the surface, the O atom will receive extra electron from the donor, leading to more filled p‐states of O atom. As a result, σ* occupancy will increase when forming the H—O bond on the surface of V_2_CO_2_. Meanwhile, the extra electron that surface O gained will also lead to less charge transfer from H to O. Therefore, the interaction between H and O will be weakened and the HER performance will be improved greatly (**Figure** **1**). To verify this point, we study the HER performance of transition metal (TM) absorbed V_2_CO_2_.

**Figure 2:**
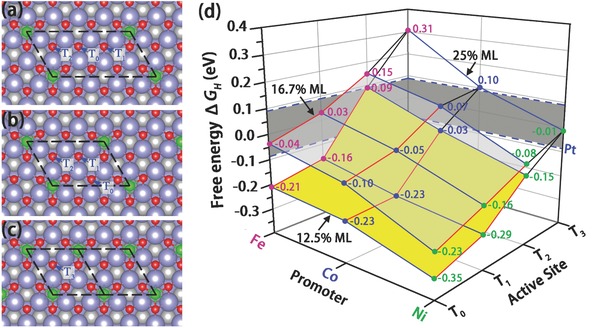
The structures and computational models of a) 12.5%, b) 16.7%, and c) 25% ML TM‐promoted V_2_CO_2_, where the blue, gray, red, and green balls present the V, C, O, and TM atoms, respectively. d) Calculated reaction free energies of hydrogen absorption Δ*G*_H_ of V_2_CO_2_ with 12.5%, 16.7%, and 25% ML TM coverage as a function of promoter and active site. The blue and red lines present the variations of Δ*G*_H_ with the changes of promoter type and active site, respectively. The yellow and transparent planes present the cases of 12.5% and 16.7% ML TM covered V_2_CO_2_, respectively, which can describe the effect of the coverage of TM on the Δ*G*_H_.

For TM‐promoted V_2_CO_2_, the TM atoms prefer to locate above the hollow sites of O_3_V_3_ and form three TM—O and three TM—V bonds with neighboring O and V atoms, respectively. The calculated binding energies (*E*_b_) between different TM atoms and V_2_CO_2_ are illustrated in Table S1 (Supporting Information), and they are all larger than 1.0 eV, indicative of the strong binding strength between TM atoms and V_2_CO_2_. The stability of TM‐promoted V_2_CO_2_ is further evaluated by ab initio molecular dynamics simulation. As shown in Figure S3 (Supporting Information), no structure reconstruction is found to occur in all of the cases under the temperature of 353 K. Even the temperature increases to 500 K, which far exceeds the typical experimental reaction temperature, the structures of the TM‐V_2_CO_2_ still remain in good shape, indicative of the high stability of TM‐V_2_CO_2_. Moreover, the interaction between the promoter atoms and surface O atoms follows the type of electron transfer, due to the relatively large electron transfer (larger than 0.7 e) from TM atoms to V_2_CO_2_ and the high formation energy of an oxygen vacancy (larger than 3.0 eV) on both the pure and TM‐promoted V_2_CO_2_ surface, which is similar to that of Pt–CeO_2_ catalyst.^27^ On the surface of TM‐promoted V_2_CO_2_, H prefers to adsorb on the top of the O atom which is not bonded with the TMs, as shown in **Figure** **2**a–c. Four different active sites can be obtained, labeled as T_0_, T_1_, T_2_, and T_3_ sites, respectively, in which “T” means the “top site of surface O atom” and the subscript is the number of surrounding V atoms that connect to both the active O atom and the promoter atom. Clearly, the larger the subscript number is, the more TM atoms near the active site. For V_2_CO_2_ with 12.5% ML coverage of TM promoter, each configuration contains five possible active sites, i.e., three T_0_, one T_1_, and one T_2_ site (Figure 2a). Similarly, the systems with 16.7% ML promoter coverage also have three kinds of active sites: T_0_, T_1_, and T_2_ sites (Figure 2b). For the case of 25% ML promoter covered V_2_CO_2_, its surface is much simple with only a T_3_ site (Figure 2c). We also examine other possible adsorption sites: (i) H adsorbs on the top site of TMs, where the calculated Δ*E*_H_ is positive, indicating it is not an active site for HER; (ii) H adsorbs on the hollow site of O_3_, however, H will diffuse to the top site of neighboring O spontaneously after full relaxation. The corresponding configurations of H adsorbed TM‐promoted V_2_CO_2_ are illustrated in Figure S4 (Supporting Information).

We select iron (Fe), cobalt (Co), and nickel (Ni) to investigate the promotional effect of different TMs on the HER performance of V_2_CO_2_ and the results are summarized in **Table** **1** and Figure 2d. As clearly shown in the table and figure, the calculated Δ*G*_H_ of the TM‐promoted V_2_CO_2_ is always larger than that of pure V_2_CO_2_, suggesting that introducing the TM onto the surface of V_2_CO_2_ can indeed weaken the H—O binding strength and thereby improve the HER performance. Most importantly, the Δ*G*_H_ at T_3_ site of 25% ML Ni‐V_2_CO_2_ (denoted as “T_3_(25% ML‐Ni)” for the sake of concision), T_0_(16.7% ML‐Fe), T_1_(16.7% ML‐Co), T_1_(16.7% ML‐Fe), and T_2_(12.5% ML‐Co) site is only −0.01, −0.04, −0.05, 0.03, and −0.03 eV, respectively. The |Δ*G*_H_| at these sites are even smaller than the Δ*G*_H_ of Pt(111) surface (≈0.09 eV),^28^ indicating the extremely high catalytic activity for HER. Other sites, such as T_3_(25% ML‐Co), T_0_(16.7% ML‐Co), T_2_(16.7% ML‐Co), T_2_(16.7% ML‐Ni), T_2_(12.5% ML‐Fe), and T_2_(12.5% ML‐Ni) have similar |Δ*G*_H_| (≈0.1 eV) to that of Pt (111) surface. Therefore, these TM‐promoted V_2_CO_2_ are expected to have HER performances that are comparable to or even better than the Pt surface.

**Table 1:** Calculated Δ*E*_H_, Δ*G*_H_, and charge transfer at different active sites of different TM‐V_2_CO_2_

System	Coverage	Active site	Δ*E*_H_ [eV]	Δ*G*_H_ [eV]	Ne [e]
Fe‐V_2_CO_2_	12.5% ML	T_0_	−0.58	−0.21	0.912
		T_1_	−0.53	−0.16	0.927
		T_2_	−0.28	0.09	0.941
	16.7% ML	T_0_	−0.41	−0.04	0.918
		T_1_	−0.34	0.03	0.933
		T_2_	−0.22	0.15	0.941
	25% ML	T_3_	−0.06	0.31	0.967
Co‐V_2_CO_2_	12.5% ML	T_0_	−0.60	−0.23	0.909
		T_1_	−0.60	−0.23	0.924
		T_2_	−0.40	−0.03	0.932
	16.7% ML	T_0_	−0.47	−0.10	0.913
		T_1_	−0.42	−0.05	0.929
		T_2_	−0.30	0.07	0.932
	25% ML	T_3_	−0.27	0.10	0.952
Ni‐V_2_CO_2_	12.5% ML	T_0_	−0.72	−0.35	0.899
		T_1_	−0.66	−0.29	0.918
		T_2_	−0.52	−0.15	0.923
	16.7% ML	T_0_	−0.60	−0.23	0.905
		T_1_	−0.53	−0.16	0.918
		T_2_	−0.45	−0.08	0.927
	25% ML	T_3_	−0.38	−0.01	0.946

Moreover, the HER catalytic activities of TM‐V_2_CO_2_ show strong dependence on the types and coverage of the promoters as well as the active sites. First, at the same level of promoter coverage and the same type of active site, the calculated Δ*G*_H_ always decreases from Fe‐ to Co‐ to Ni‐V_2_CO_2_. For example, at the T_3_ site of V_2_CO_2_ with 25% ML promoter coverage, the calculated Δ*G*_H_ is 0.31 eV for Fe‐V_2_CO_2_, 0.10 eV for Co‐V_2_CO_2_, and −0.01 eV for Ni‐V_2_CO_2_, respectively. Second, for a given active site and a given promoter type, the calculated Δ*G*_H_ always increases with the increase of the promoter coverage. This can be seen vividly from Figure 2d that the yellow plane which presents the case of 12.5% ML promoter coverage is always under the transparent plane which describes the situation of 16.7% ML promoter coverage. Third, the HER activity is also sensitive to the active site. As displayed in Figure 2d, the calculated Δ*G*_H_ for 12.5% ML Ni‐promoted V_2_CO_2_ is −0.32 eV at T_0_ site, and it increases to −0.27 and −0.12 eV at T_1_ and T_2_ site, while the T_3_ site has the largest Δ*G*_H_, respectively. For the case of Fe‐ and Co‐promoted systems, similar tendency is also observed. That is, Δ*G*_H_ of T_0_ site is the lowest, followed by that of T_1_, T_2_, and T_3_ sites. Therefore, we can conclude, for a certain promoter, the H binding strength will decrease monotonously from T_0_ to T_1_ to T_2_ and to T_3_ site, leading to the increase of Δ*G*_H_ gradually. These promoter type, coverage, and active site dependent behaviors offer us flexibility for modulating the HER performance of the systems and the optimal coverage for Fe‐, Co‐, and Ni‐V_2_CO_2_ is ≈16.7% ML, 16.7%–25% ML, and ≈25% ML, respectively. Moreover, at these optimal coverages, all the active sites present high HER activity, which is actually important, since it is hard to control the reaction occurred at certain sites in real experiments.

**Figure 3:**
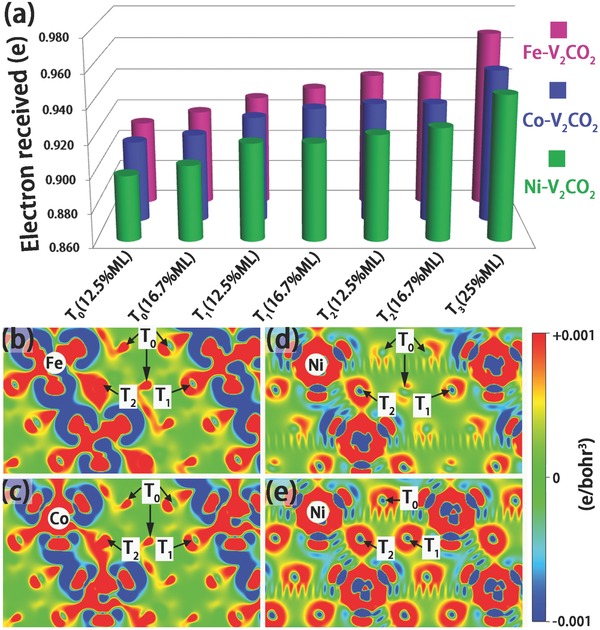
a) The number of electron that different active sites received. Difference charge density of b) 12.5% ML Fe‐promoted, c) 12.5% ML Co‐promoted, d) 12.5% ML Ni‐promoted, and e) 16.7% ML Ni‐promoted V_2_CO_2_, respectively.

The promotional effect of TM on HER performance of V_2_CO_2_ can be ascribed to the significant charge transfer between promoter atom and surface O atoms. Through Bader charge analysis, for pure V_2_CO_2_, each surface O atom gains about 0.895 e from surrounding V atoms (see Table 1). For the TM promoted V_2_CO_2_, the electrons that surface O atoms received (*N_e_*) range from 0.899 to 0.967 e (Table 1 and **Figure** **3**a), larger than those in pure V_2_CO_2_. As discussed above, these extra electrons will lead to more p‐states of O atom and increase the σ* occupancy in TM promoted V_2_CO_2_. As a result, the H—O bond of TM‐V_2_CO_2_ is weakened, as compared with that in pure V_2_CO_2_ and eventually increases Δ*G*_H_. Moreover, different TM promoters cause different charge transfer, which is determined by the intrinsic electronegativity of the TM. As the electronegativity order of these three TMs follows: Fe (1.83) < Co (1.88) < Ni (1.92), Fe will provide the largest charge transfer to O atom, followed by Co and Ni. As a result, at 12.5% ML TM coverage, the charge near the active sites of Fe‐promoted V_2_CO_2_ is the densest, followed by Co‐ and Ni‐promoted systems (Figure 3b–d). For other TM coverage, similar tendency is observed, as shown in Table 1 and Figure 3a. Correspondingly, the H—O bond in Fe‐promoted V_2_CO_2_ is always weaker than that in Co‐ and Ni‐promoted systems. Therefore, the Δ*G*_H_ in Ni‐V_2_CO_2_ is always the smallest, followed by Co‐V_2_CO_2_ and Fe‐V_2_CO_2_. As more TM atoms surround the active site (from T_0_ to T_1_ to T_2_ to T_3_), the number of received electrons of O atom increases for all TM‐promoted systems. As a result, the H—O binding strength is attenuated and Δ*G*_H_ is thus enhanced. With the increase of Ni coverage from 12.5% ML (Figure 3d) to 16.7% ML (Figure 3e), the charge density at the same type of active site always increases. For other promoters, the received charge of a certain site at a lower TM coverage is also less than that at a higher TM coverage (Table 1 and Figure 3a). Therefore, the H—O bond is weakened and Δ*G*_H_ is augmented with the increase of TM coverage. These results are in perfect accord with the promoter type, coverage and active site dependent behaviors discussed above, which shed light on the nature of the promotional effects of transition metals on the catalytic activity.

A complete HER is a multistep reaction. The first step is the hydrogen adsorption, named Volmer reaction, in which a proton gains an electron from the surface of catalyst or electrode to form adsorbed hydrogen. The subsequent step has two possibilities: one is the homolytic Tafel reaction, 2H_ad_ → H_2_; the other is the heterolytic Heyrovsky reaction, H_ad_ + H^+^ + e^−^ → H_2_. The pathway of the second step is strongly dependent on the inherent properties of the catalyst or electrode. Above discussion belongs to Volmer reaction. To give a comprehensive understanding of the HER process of TM‐V_2_CO_2_, we further explore possibilities of Tafel mechanism or Heyrovsky mechanism. In fact, all the active sites that present high activity for Volmer reaction will maintain their catalytic activity for Heyrovsky reaction. Therefore, the second step of HER on the surface of TM‐promoted V_2_CO_2_ can follow the Heyrovsky mechanism. To verify whether the reaction can also follow the Tafel mechanism, we select 16.7% Fe‐ and Co‐promoted V_2_CO_2_ as prototypes and calculate the Δ*G*_H_ for the adsorption of the second hydrogen. As shown in Figure S5 (Supporting Information), with the adsorption of the second hydrogen, the Δ*G*_H_ greatly increases from −0.04 and −0.10 eV to 0.42 and 0.37 eV for Fe‐ and Co‐V_2_CO_2_, respectively, indicating that the second HER step of TM‐promoted V_2_CO_2_ may not follow the Tafel mechanism. To further verify this, the activation barriers following Tafel mechanism are calculated. As shown in Figure S6 (Supporting Information), relative high energy barriers need to be overcome, i.e., 2.13 and 2.22 eV for Fe‐ and Co‐V_2_CO_2_, respectively. Therefore, we can conclude that the HER on the surface of Fe‐ and Co‐promoted V_2_CO_2_ follows the Heyrovsky mechanism rather than the Tafel mechanism.

Strain engineering has been proved to be an efficient way to tune the physical and chemical properties of 2D materials, including MXenes,^29^, ^30^, ^31^ which may have influence on the HER performance as well. Moreover, the real HER experiments are generally very complicated. Materials may suffer deformations and form curved surfaces, in which the concave and convex can be regarded as suffering compressive and tensile strain, respectively. We select 12.5% ML TM covered V_2_CO_2_ which contains T_0_, T_1_, and T_2_ sites and the 25% ML TM covered V_2_CO_2_ which contains T_3_ site as representatives to study the strain influence on the HER performance of the TM‐promoted V_2_CO_2_. Here, the biaxial strain is considered and defined as *ε =* Δ*a*/*a*_0_, where the *a*_0_ and Δ*a* +*a*_0_ are the lattice constants of the unstrained and strained supercells, respectively. Thus, the positive or negative values of *ε* correspond to the tensile or compressive strain, respectively.

**Figure 4:**
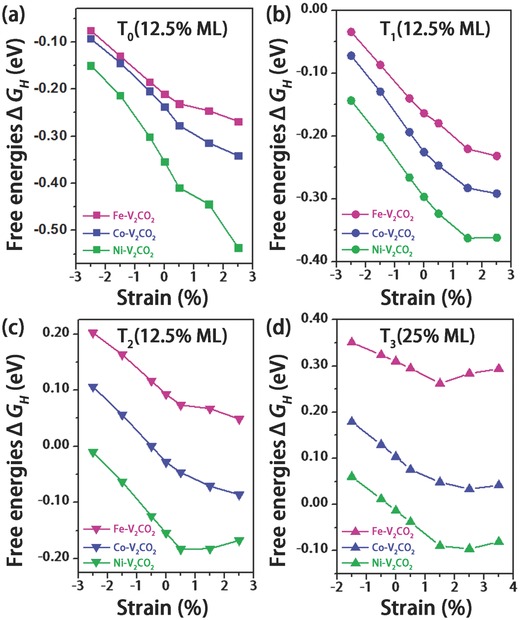
Calculated free energy for hydrogen adsorption Δ*G*_H_ at a) T_0_ (12.5% ML), b) T_1_ (12.5% ML), c) T_2_ (12.5% ML), and d) T_3_ (25% ML) sites as a function of strain. The purple, blue, and green lines represent Fe‐, Co‐, and Ni‐promoted systems, respectively.

**Figure** **4** presents the reaction free energy for hydrogen adsorption at T_0_, T_1_, and T_2_ sites of V_2_CO_2_ with the promoter coverage of 12.5% ML and at T_3_ sites of V_2_CO_2_ with the coverage of 25% ML as a function of strain. A general tendency is observed, that is, the Δ*G*_H_ monotonously decreases with the increase of *ε*. Consequently, for strong H binding strength systems that have relatively negative Δ*G*_H_, the compressive strain can improve their HER performance. For example, the Δ*G*_H_ of T_0_ (12.5% ML‐Co) and T_1_ (12.5% ML‐Fe) sites is −0.24 and −0.16 eV at unstrained state and reduces to −0.09 and −0.03 eV under −2.5% strain, respectively. On the contrary, the tensile strain is an efficient way to improve the HER performance of weak H binding strength systems, such as T_3_(25% ML‐Co) site, whose Δ*G*_H_ will decrease from 0.10 to 0.03 eV when applying a 2.5% tensile strain on it. Moreover, the HER catalytic performance of V_2_CO_2_ can even be tuned to reach the optimal Δ*G*_H_ of 0 eV by applying a biaxial strain, i.e., T_2_(12.5% ML‐Co) under −0.5% strain and T_3_(25% ML‐Ni) under −0.27% strain. Most interestingly, for highly active sites such as T_2_(12.5% ML‐Co) and T_3_(25% ML‐Ni) sites, the calculated |Δ*G*_H_| is always smaller than 0.1 eV within a relatively wide range of strain, indicating their highly catalytic stability when used in real condition.

The strain engineering HER performance of TM‐promoted V_2_CO_2_ can be profoundly understood in light of the partial density of states of surface O under different strain. As clearly illustrated in Figures S7–S18 (Supporting Information), the p‐orbital DOS of surface O atom in TM‐promoted V_2_CO_2_ shows an upward shift with the increase of *ε*. These upward shifts will lead to fewer filled p‐states,^32^ and less σ* occupancy with the increase of *ε*; as a consequence, the binding strength of H—O bond will be enhanced and the Δ*G*_H_ is accordingly decreased.

## 3 Conclusion

In summary, we study the HER performance of fully oxidized vanadium carbides V_2_CO_2_ with and without the promotion of transition metals within the framework of first‐principle calculations. Our calculations show that pure V_2_CO_2_ is not an ideal catalyst for HER, while it can be engineered to be an excellent HER catalyst by introducing the TM atoms onto the surface. The influences of the TM promoter type, coverage, and the active site on the HER performance of V_2_CO_2_ are further explored in details and ≈16.7% ML Fe‐promoted, 16.7%–25% ML Co‐promoted, ≈25% ML Ni‐promoted systems are found to be the best catalysts for HER activity with the optimal Δ*G*_H_ of ≈0 eV. Moreover, these TM‐promoted catalysts show good catalytic stability and can be further modulated by applying external strain as well. It is worth pointing out that assembling various TM onto the surfaces of materials can be easily realized in experiment, while the size and coverage can also be controlled by adjusting the ratio of reactants, react time, type, and amount of surfactant.^33^, ^34^ Therefore, these TM promoted V_2_CO_2_ are expected to be a kind of easy‐synthesized and highly active catalyst for HER. In short, the findings unveiled here would open a new window for the application of 2D MXenes and for the development of cost‐effective alternatives to Pt in HER.

## 4 Experimental Section

All first‐principle calculations were performed by using projector augmented wave method^35^ as implemented in the Vienna ab initio simulation package.^36^, ^37^ The generalized gradient approximation in the Perdew–Burke–Ernzerhof form^38^, ^39^ and a cut‐off energy of 600 eV for plane‐wave basis set were adopted. The convergence threshold was 10^−5^ eV for energy and 0.02 eV Å^−1^ for force, respectively. To avoid the interaction between two periodic units, a vacuum space at least 20 Å was used. Both non‐polarized and spin‐polarized calculations were employed to determine the ground state structures. Supercells consisting of 4 × 2 × 1, 3 × 2 × 1, and 2 × 2 × 1 unit cells of V_2_CO_2_ ML were used for 12.5% ML, 16.7% ML, and 25% ML hydrogen adsorbed or promoter covered systems, respectively, as shown in Figure S1 in the Supporting Information. The corresponding Brillouin zone was sampled by Monkhorst–Pack k‐point mesh of 4 × 8 × 1, 5 × 9 × 1, and 9 × 9 × 1, respectively.

The HER catalytic activity of materials can be evaluated by the reaction free energy of hydrogen adsorption (Δ*G*_H_),^6^, ^28^ defined as

$$\Delta G_{H}=\Delta E_{H}+\Delta E_{\mathrm{ZPE}}-T\Delta S_{H}$$

where Δ*E*_H_ is the hydrogen adsorption energy

$$\Delta E_{H}=E_{\left(\mathrm{System}+H\right)}-E_{\left(\mathrm{System}\right)}-\frac{1}{2}E_{H_{2}}$$

in which Δ*E*_(System + H)_ and Δ*E*_(System)_ are the energies of V_2_CO_2_ systems with and without H adsorption, respectively. Δ*E*_ZPE_ and Δ*S*_H_ are the zero‐point energy difference and the entropy difference between the adsorbed and the gas phase, respectively. The Δ*E*_ZPE_ can be obtained via vibrational frequency calculation. The Δ*S*_H_ can be regarded as $\Delta S_{H}≅\frac{1}{2}S_{H_{2}}^{0}$ due to the fact that the vibrational entropy in the adsorbed state is small according to previous studies.^28^, ^40^ The optimal value for HER is Δ*G*_H_ = 0, which means that the smaller values of |Δ*G*_H_|, the better HER performance of materials.

## Supporting information

As a service to our authors and readers, this journal provides supporting information supplied by the authors. Such materials are peer reviewed and may be re‐organized for online delivery, but are not copy‐edited or typeset. Technical support issues arising from supporting information (other than missing files) should be addressed to the authors.

Supplementary
