# Supplementary material for: Transition Metal‐Promoted V2CO2 (MXenes): A New and Highly Active Catalyst for Hydrogen Evolution Reaction
Source: Adv Sci (Weinh). 2016 Jun 28;3(11):1600180. doi: 10.1002/advs.201600180 (PMC5102657; doi:10.1002/advs.201600180)
Supplement: Supplementary file 1 — Supplementary [file ADVS-3-0d-s001.pdf]

Copyright WILEY-VCH Verlag GmbH & Co. KGaA, 69469 Weinheim, Germany, 2016.

ADVANCED SCIENCE
Open Access

Supporting Information

for *Adv. Sci.,* DOI: 10.1002/advs.201600180

Transition Metal-Promoted V_2CO_2 (MXenes): A New and Highly Active Catalyst for Hydrogen Evolution Reaction

*Chongyi Ling, Li Shi, Yixin Ouyang, Qian Chen, and Jinlan Wang**

Supporting Information

Transition metal-promoted V_2CO_2 (MXenes): A New and Highly Active Catalyst for Hydrogen Evolution Reaction

*Chongyi Ling, Li Shi, Yixin Ouyang, Qian Chen and Jinlan Wang**

Figures

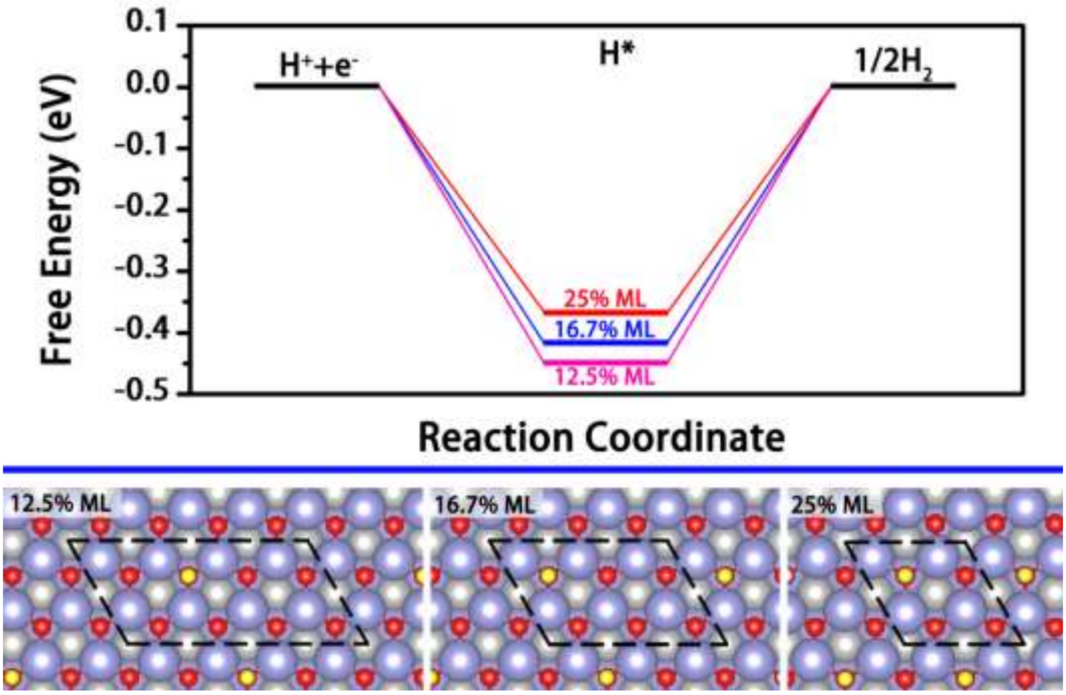

Figure S1. The calculated free energies for hydrogen adsorption as a function of H-coverage and the computational models of V_2CO_2 with different H coverage. The blue, gray, red and yellow balls present the V, C, O and H atoms, respectively.

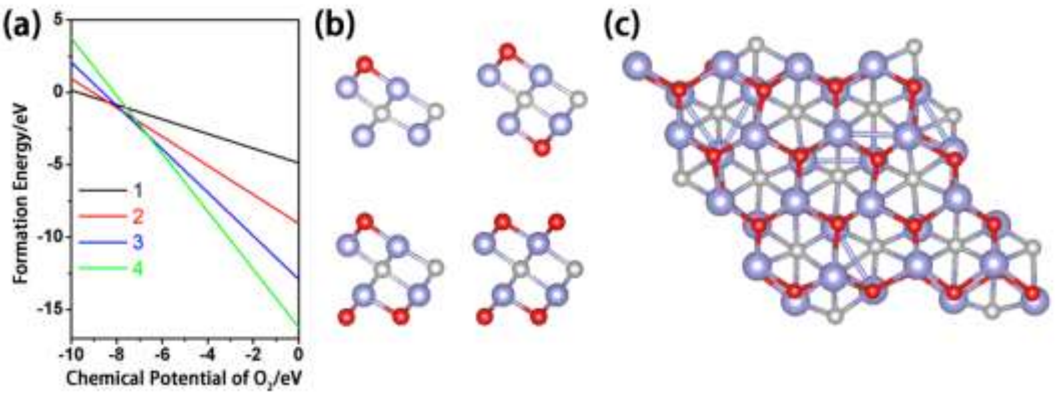

Figure S2. (a) Formation energies of a 2 × 1 V_2C supercell consisting of 1-4 oxygen atoms as a function of the oxygen (gas) chemical potential in the left panel. (b) The corresponding structures from the side view were illustrated in the right panel, in which the blue, gray and red balls present the V, C and O atoms, respectively. (c) Snapshots of atomic configurations of V_2CO_2 at the end of AIMD simulations under 1000K. The model we used is a supercell consist of 4 × 4 unit cells of V_2CO_2. The ab initio molecular dynamics calculations were typically run for 5 ps, with a time step of 2 fs.

The formation energies E_f were calculated by using the following equation:

$$E_f = E_{V_2C-O} - E_{V_2C} - n/2E_{O_2} - n/2\mu_{O_2}$$

where E_{V_2C-O}, E_{V_2C} and E_{O_2} are the energies of O adsorbed V_2C, pure V_2C and O_2 molecule, respectively; n is the number of adsorbed O and μ_{O_2} is the chemical potential of O_2 gas.

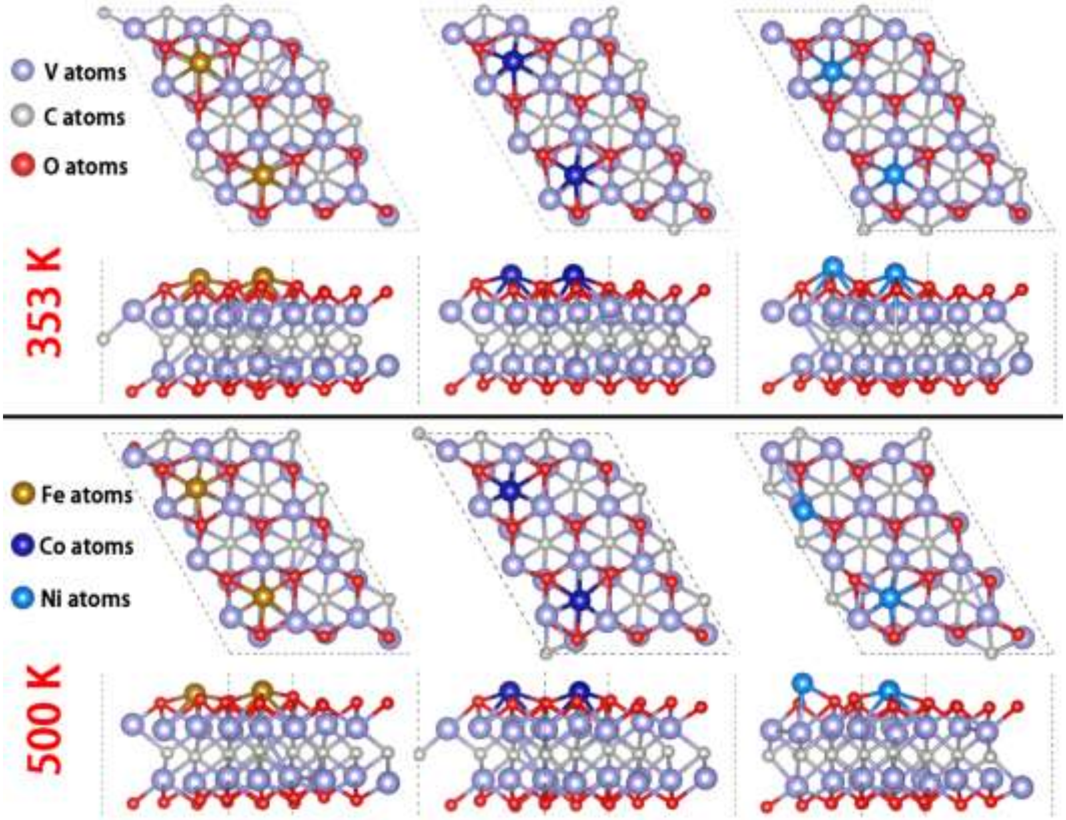

Figure S3. Snapshots of atomic configurations of TM-promoted V_2CO_2 at the end of AIMD simulations under different temperature. The ab initio molecular dynamics calculations were typically run for 5 ps, with a time step of 2 fs.

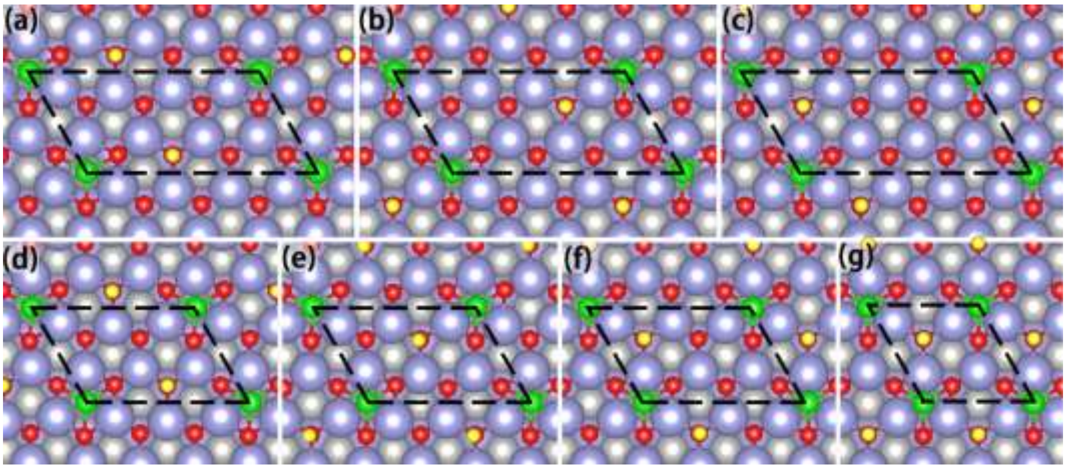

Figure S4. The structures of H adsorbed TM-V_2CO_2. (a), (b) and (c) show H adsorbed at T_0, T_1 and T_2 sites of 12.5% ML TM covered V_2CO_2, respectively. (d), (e) and (f) present H adsorbed at T_0, T_1 and T_2 sites of 16.7% ML TM promoted V_2CO_2. (g) is the structure of 25% ML TM covered V_2CO_2 with the adsorption of H. The blue, gray, red, green and yellow balls present V, C, O, TM (Fe, Co, Ni) and H atoms, respectively.

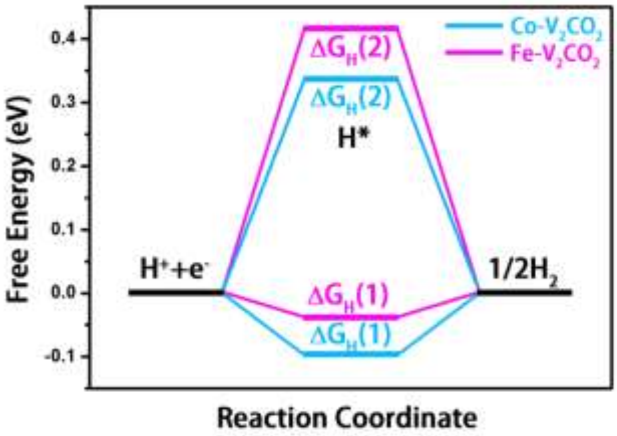

Figure S5. Calculated free energy diagram for the adsorption of two hydrogen atoms on the surface of 16.7% ML Fe- and Co-promoted V_2CO_2.

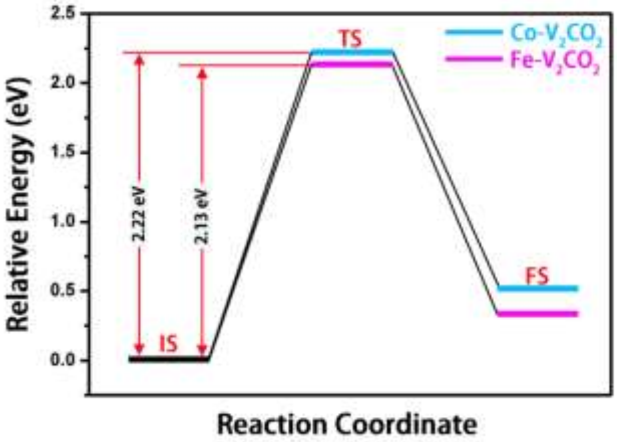

Figure S6. Calculated activation barrier during HER follows Tafel mechanism on the surface of 16.7% ML Fe- and Co-promoted V_2CO_2.

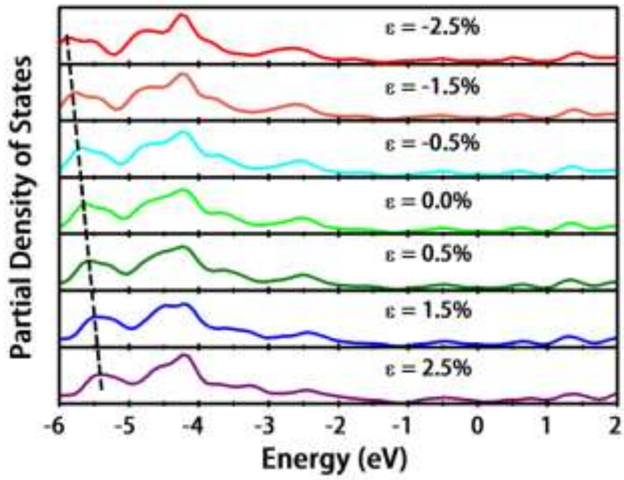

Figure S7. Partial density of states (PDOS) of O atom p orbital at T_0 site of 12.5% ML Fe-promoted V_2CO_2 under various strain. Only the majority spin states were shown as the minority spins present similar tendency.

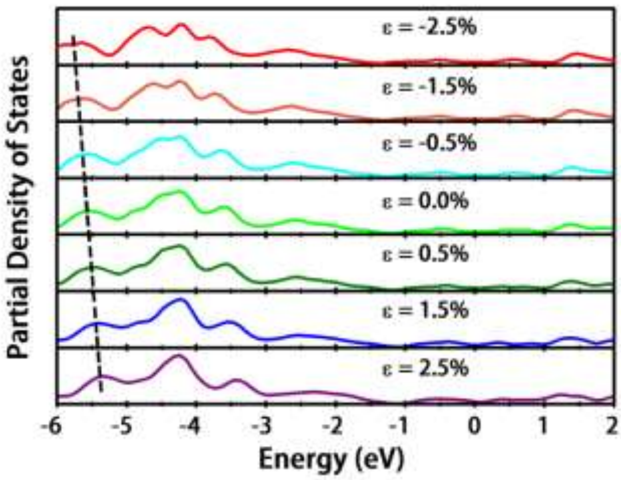

Figure S8. PDOS of O atom p orbital at T_1 site of 12.5% ML Fe-promoted V_2CO_2 under various strain. Only the majority spin states were shown as the minority spins present similar tendency.

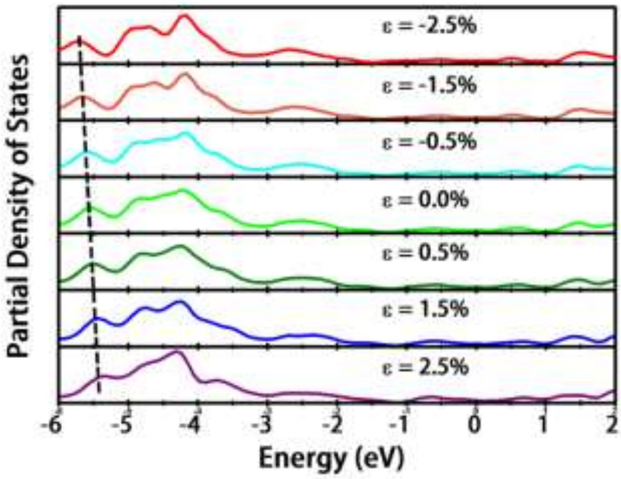

Figure S9. PDOS of O atom p orbital at T2 site of 12.5% ML Fe-promoted V_2CO_2 under various strain. Only the majority spin states were shown as the minority spins present similar tendency.

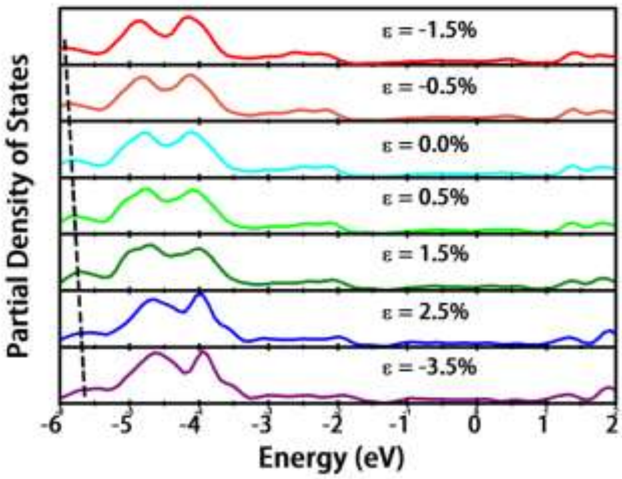

Figure S10. PDOS of O atom p orbital at T3 site of 25% ML Fe-promoted V_2CO_2 under various strain. Only the majority spin states were shown as the minority spins present similar tendency.

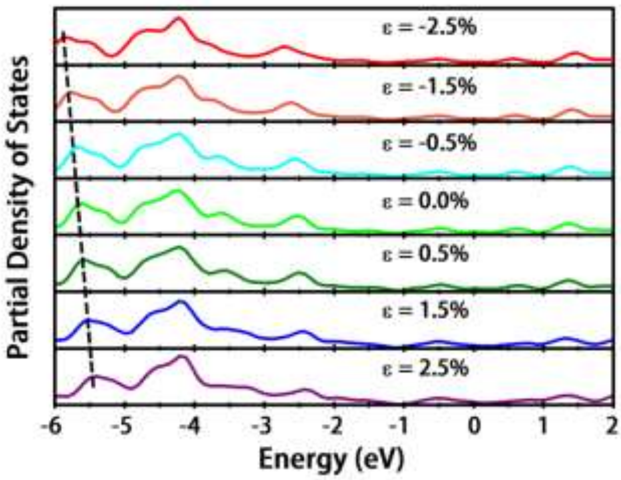

Figure S11. PDOS of O atom p orbital at T_0 site of 12.5% ML Co-promoted V_2CO_2 under various strain. Only the majority spin states were shown as the minority spins present similar tendency.

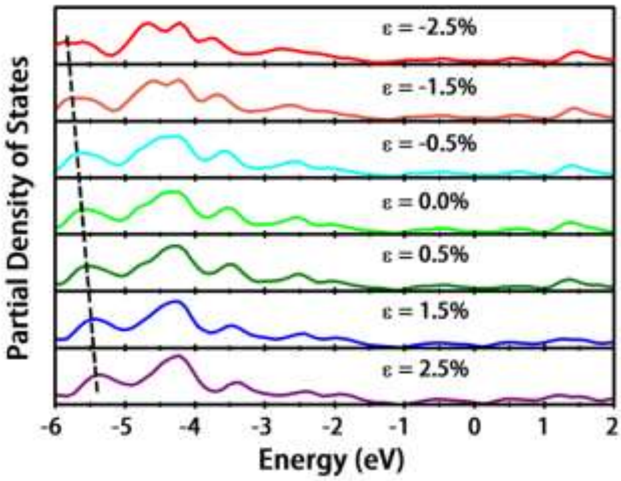

Figure S12. PDOS of O atom p orbital at T_1 site of 12.5% ML Co-promoted V_2CO_2 under various strain. Only the majority spin states were shown as the minority spins present similar tendency.

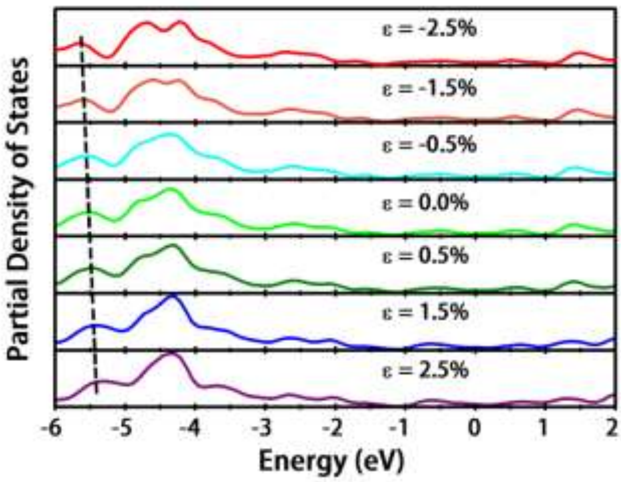

Figure S13. PDOS of O atom p orbital at T_2 site of 12.5% ML Co-promoted V_2CO_2 under various strain. Only the majority spin states were shown as the minority spins present similar tendency.

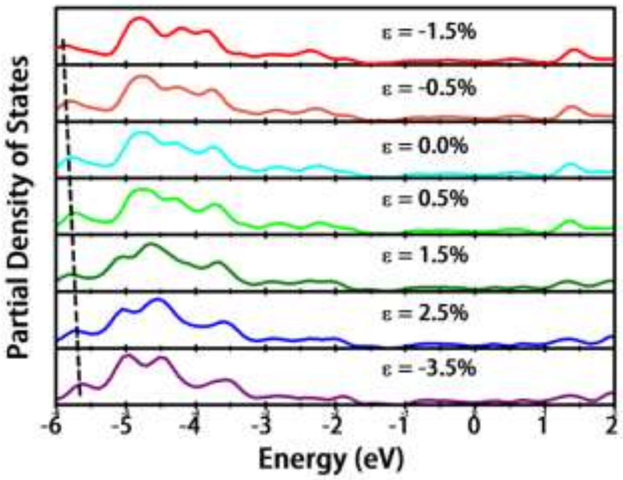

Figure S14. PDOS of O atom p orbital at T_3 site of 25% ML Co-promoted V_2CO_2 under various strain. Only the majority spin states were shown as the minority spins present similar tendency.

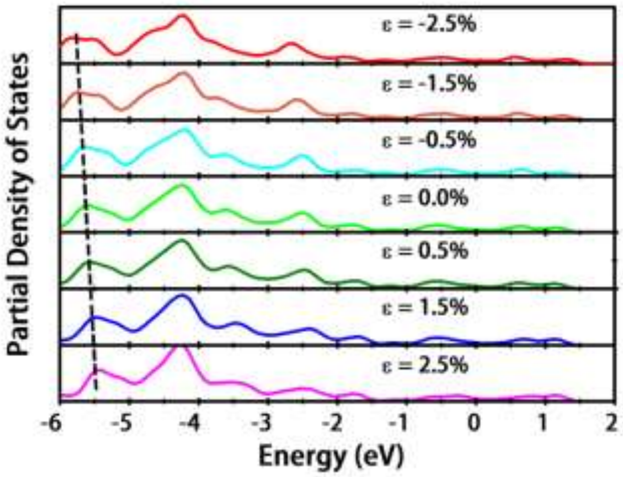

Figure S15. PDOS of O atom p orbital at T_0 site of 12.5% ML Ni-promoted V_2CO_2 under various strain.

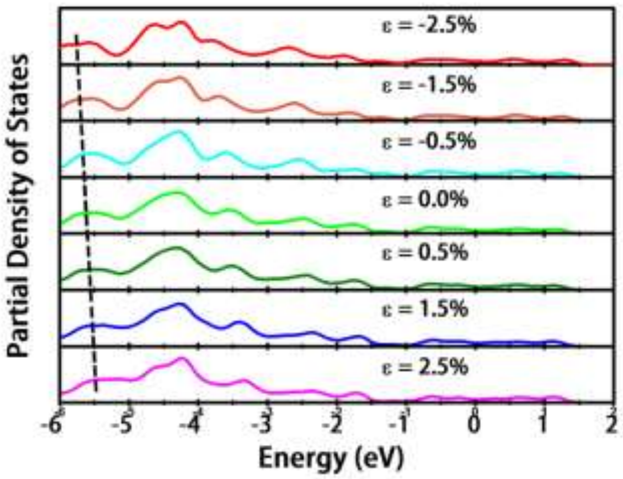

Figure S16. PDOS of O atom p orbital at T_1 site of 12.5% ML Ni-promoted V_2CO_2 under various strain.

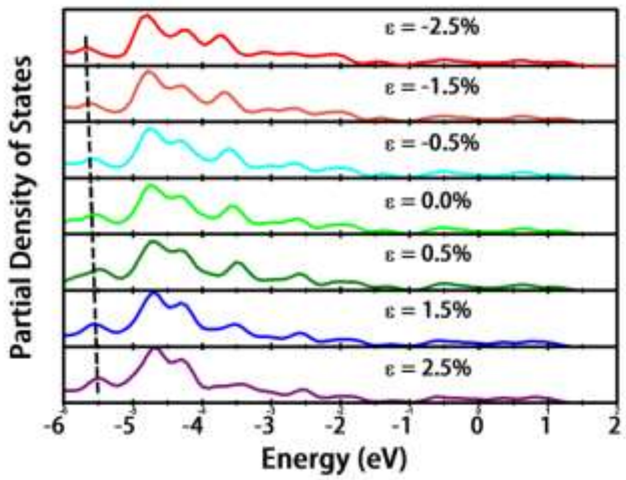

Figure S17. PDOS of O atom p orbital at T_2 site of 12.5% ML Ni-promoted V_2CO_2 under various strain.

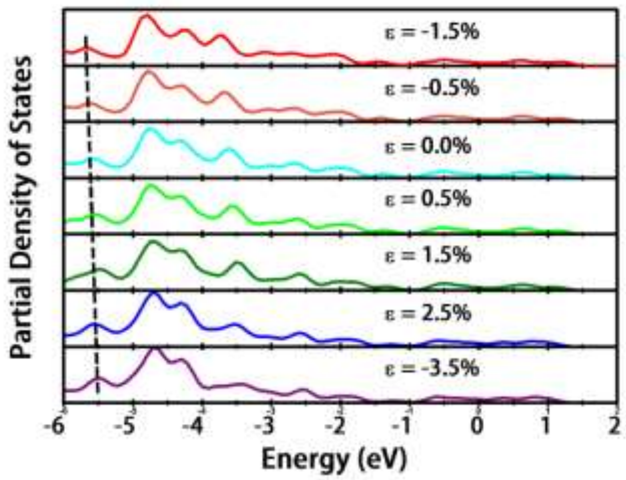

Figure S18. PDOS of O atom p orbital at T_3 site of 25% ML Ni-promoted V_2CO_2 under various strain.

Tables

Table S1. The binding energy (in eV) between different transition metals and V_2CO_2.

TMs \ E_b \ Coverage	**12.5%**	**16.7%**	**25%**
**Fe**	-1.00	-1.04	-1.21
**Co**	-1.57	-1.58	-1.75
**Ni**	-1.64	-1.67	-1.75

The binding energy is calculated by the following equation:

$$E_b = E_{(V_2CO_2+nTM)} - E_{V_2CO_2} - nE_{TM}$$

where the $E_{(V_2CO_2+nTM)}$ and $E_{V_2CO_2}$ are the energies of V_2CO_2 with and without the adsorption of TM atoms, respectively; E_{TM} is the energies of TM atoms in their metal crystals and n is the number of adsorbed TM atoms.
